# Comparative Analysis of Age-Related Changes in Lacrimal Glands and Meibomian Glands of a C57BL/6 Male Mouse Model

**DOI:** 10.3390/ijms21114169

**Published:** 2020-06-11

**Authors:** Chang Ho Yoon, Jin Suk Ryu, Ho Sik Hwang, Mee Kum Kim

**Affiliations:** 1 Department of Ophthalmology, Seoul National University College of Medicine, Seoul 03080, Korea; ifree7@gmail.com; 2 Laboratory of Ocular Regenerative Medicine and Immunology, Seoul Artificial Eye Center, Seoul National University Hospital Biomedical Research Institute, Seoul 03080, Korea; enter2357@naver.com; 3 Department of Ophthalmology, Seoul National University Hospital, Seoul 03080, Korea; 4 Department of Ophthalmology, College of Medicine, The Catholic University of Korea, Seoul 07345, Korea

**Keywords:** dry eye, aging, lacrimal glands, meibomian glands, inflammation, oxidative stress, senescence

## Abstract

It is not known how biological changes in the lacrimal (LGs) and meibomian (MGs) glands contribute to dry eye disease (DED) in a time-dependent manner. In this study, we investigated time-sequenced changes in the inflammation, oxidative stress, and senescence of stem cells in both glands of an aging-related DED mouse model. Eight-week (8W)-, one-year (1Y)-, and two-year (2Y)-old C57BL/6 male mice were used. MG areas of the upper and lower eyelids were analyzed by transillumination meibography imaging. The number of CD45^+^, 8-OHdG^+^, Ki-67^+^, and BrdU^+^ cells was compared in both glands. Increased corneal staining and decreased tear secretion were observed in aged mice. The MG dropout area increased with aging, and the age-adjusted MG area in lower lids was negatively correlated with the National Eye Institute (NEI) score. Increased CD4^+^ interferon (IFN)-γ^+^ cells in LGs were found in both aged mice. An increase in 8-OHdG^+^ cells in both glands was evident in 2Y-old mice. Reduced Ki-67^+^ cells, but no change in CD45^+^ cells, was observed in the MGs of 1Y-old mice. Increased BrdU^+^ cells were observed in the LGs of aged mice. This suggests that age-dependent DED in C57BL/6 mice is related to inflammation of the LGs, the development of MG atrophy, and oxidative stress in both glands.

## 1. Introduction

Aging is a biologically complex process associated with cellular senescence and dysregulation of the immune, endocrine, and metabolic systems, resulting in multiple organ pathologies [1,2]. Chronic oxidative stress affects the aging process by modifying interactions of these systems [2]. Given that dry eye prevalence increases with age [3], the aging process appears to significantly affect dry eye disease (DED).

The aging-dependent changes in the ocular surface with DED are related to biological changes in the lacrimal glands (LGs) and meibomian glands (MGs). Although the pathogenesis of aging-associated DED has not yet been fully understood, recent studies have shown that autoimmune changes or oxidative stress in the LGs and acinar cell atrophies in the MGs occur with aging [4,5,6,7,8].

However, it is not known how biological changes in both the LGs and MGs contribute to DED in a time-dependent manner. With regard to the senescence of stem cells, oxidative stress, and gland inflammation, it has not been determined which process contributes to the profound ocular surface changes with aging when the two glands are compared to each other over time. Therefore, we aimed to investigate the time-sequenced changes in the inflammation, oxidative stress, and senescence of progenitor cells in both the LGs and MGs, as well as compare the changes between them in an age-related DED C57BL/6 mouse model.

## 2. Results

### 2.1. DED Was Established in Aged C57BL/6 Male Mice

**Figure 1:**
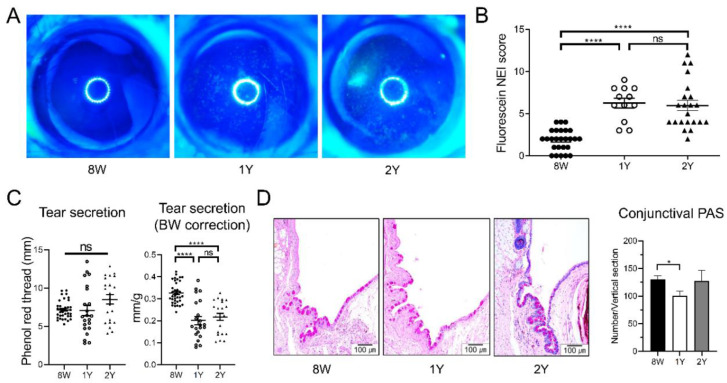
(**A**) Representative images of corneal fluorescein staining. (**B**) The National Eye Institute (NEI) corneal staining score was significantly higher in one-year (1Y)- and two-year (2Y)-old mice than in eight-week (8W)-old mice (all *p* < 0.0001; Kruskal–Wallis test followed by Dunn’s post hoc test; *n* = 26, 12, and 22 for 8W-, 1Y-, and 2Y-old mice, respectively). (**C**) Tear secretion did not change over time (Kruskal–Wallis test). However, when the level was normalized by body weight (BW), the normalized value was lower in 1Y- and 2Y-old mice than in 8W-old mice (all *p* < 0.0001; one-way ANOVA followed by Tukey’s post hoc test; *n* = 36, 22, and 22 for 8W-, 1Y-, and 2Y-old mice, respectively). (**D**) Representative photographs of periodic acid–Schiff (PAS) staining of the inferior conjunctival fornix (sagittal section; 200×). The number of goblet cells was significantly lower in 1Y-old mice than in 8W-old mice (*p* = 0.043; one-way ANOVA followed by Tukey’s post hoc test; *n* = 12, 6, and 3 for 8W-, 1Y-, and 2Y-old mice, respectively). Ns, not significant; * *p* < 0.05 and **** *p* < 0.0001. Data are presented as means ± standard error.

Corneal punctate epithelial erosion was higher in the aged (1-year (1Y)-old and 2-year (2Y)-old) mice than in eight-week (8W)-old mice. The mean (±SD) corneal staining scores of 8W-, 1Y-, and 2Y-old mice were 1.88 ± 1.28, 6.25 ± 2.01, and 5.95 ± 2.85, respectively. The scores of the 1Y- and 2Y-old mice groups were significantly higher than those of the 8W-old mice (all *p* < 0.0001; Kruskal–Wallis test followed by Dunn’s post hoc test); however, no difference was observed between 1Y- and 2Y-old mice (Figure 1A,B). Tear secretion was not different among the groups; however, when the body weight was adjusted, the normalized value was significantly lower in aged mice than in 8W-old mice (Figure 1C; all *p* < 0.0001; one-way ANOVA followed by Tukey’s post hoc test). Periodic acid–Schiff (PAS) staining of the conjunctiva showed that the number of goblet cells was significantly lower in 1Y-old mice than in 8W-old mice (Figure 1D; *p* = 0.043; one-way ANOVA followed by Tukey’s post hoc test). However, the number of goblet cells in 2Y-old mice was not different from the number in 8W- or 1Y-old mice. Given these results, DED was fully established at one year of age in C57BL/6 male mice.

### 2.2. MG Dropout Area Increased with Aging, and Age-Adjusted Lower MG Area Was Negatively Correlated with the Severity of Corneal Epithelial Erosion

**Figure 2:**
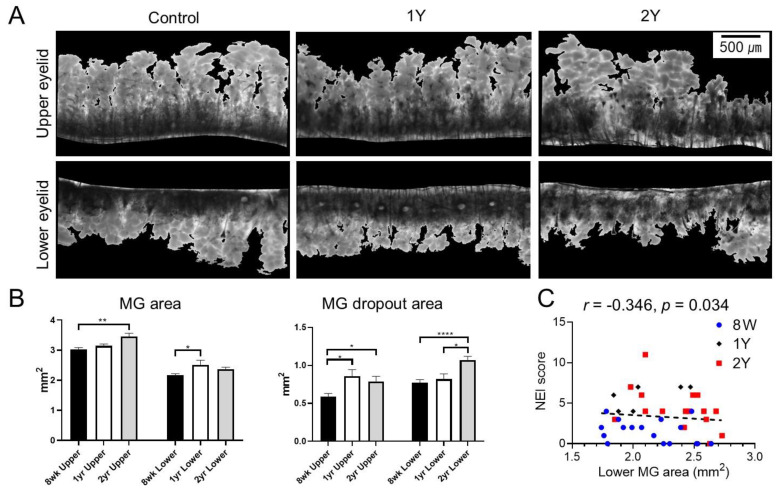
(**A**) Representative transillumination meibography images. (**B**) Meibomian gland (MG) areas of the upper eyelid in 2Y-old mice (*p* = 0.004; Kruskal–Wallis test followed by Dunn’s post hoc test) and lower eyelid in 1Y-old mice (*p* = 0.021; one-way ANOVA followed by Tukey’s post hoc test) were larger than those in 8W-old mice. MG dropout areas of upper eyelids were larger in 1Y- and 2Y-old mice than 8W-old mice (*p* = 0.023 and *p* = 0.02, respectively; Kruskal–Wallis test followed by Dunn’s post hoc test). MG dropout areas of lower eyelids were larger in 2Y-old mice than 8W- and 1Y-old mice (*p <* 0.0001 and *p* = 0.029, respectively; one-way ANOVA followed by Tukey’s post hoc test). (**C**) The age-adjusted MG area of the lower eyelid was negatively correlated with the National Eye Institute (NEI) score (*r* = −0.346; *p* = 0.034; partial correlation analysis). * *p* < 0.05, ** *p* < 0.01, and **** *p* < 0.0001. Data are presented as means ± standard error.

Transillumination meibography showed less dense MG in aged mice (1Y- and 2Y-old mice) than in 8W-old mice (Figure 2A). The MG areas in 2Y-old mice were larger than those in 8W-old mice (*p* = 0.004; Kruskal–Wallis test followed by Dunn’s post hoc test). The MG areas of the lower eyelid in 1Y-old mice were larger than those in 8W-old mice (*p* = 0.021; one-way ANOVA followed by Tukey’s post hoc test). The MG dropout areas increased with aging. Upper MG dropout areas in 1Y- and 2Y-old mice were larger than those in 8W-old mice (*p* = 0.023 and *p* = 0.02, respectively; Kruskal–Wallis test followed by Dunn’s post hoc test) and lower MG dropout areas in 2Y-old mice were larger than those in 8W- and 1Y-old mice (*p <* 0.0001 and *p* = 0.029, respectively; one-way ANOVA followed by Tukey’s post hoc test). Interestingly, the lower MG area was negatively correlated with the corneal staining score after age adjustment (*r* = −0.346; *p* = 0.034; partial correlation analysis; Figure 2C). However, upper MG areas and upper MG dropout areas did not show any correlation to the corneal staining score after age adjustment in this mouse model (*r* = −0.213, *p* = 0.200 and *r* = −0.195, *p* = 0.240, respectively; partial correlation analysis).

### 2.3. Aging-Dependent Inflammation Was Not Evident, but Decreased Potential of Differentiation Was Suspected in the MGs

**Figure 3:**
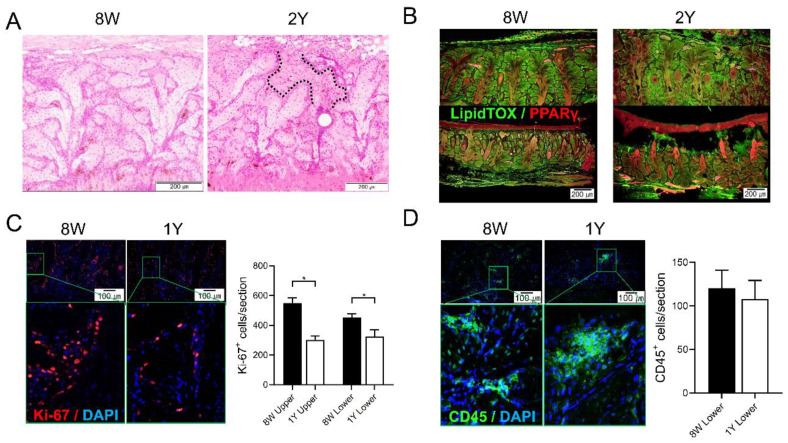
(**A**) Representative images of coronal sections of the MGs stained with hematoxylin-eosin (H&E). The area between the dotted lines indicates suspicious MG dropout (200×). (**B**) Representative images of coronal sections of the MGs stained with LipidTOX and PPARγ immunofluorescence (100×). (**C**) The expression of Ki67^+^ cells in both MGs of the upper and lower eyelids was significantly reduced in 1Y-old mice compared to 8W-old mice (*p* = 0.036 by the Mann–Whitney test and 0.035 by the independent *t*-test, respectively; 200×). (**D**) The expression of CD45^+^ cells in the MGs of the lower eyelid was not different between 8W- and 1Y-old mice (*p* = 0.571; Mann–Whitney test; 200×). All images were obtained from the coronal section. *n* = 5 and 3 for 8W- and 1Y-old mice, respectively. * *p* < 0.05.

**Figure 4:**
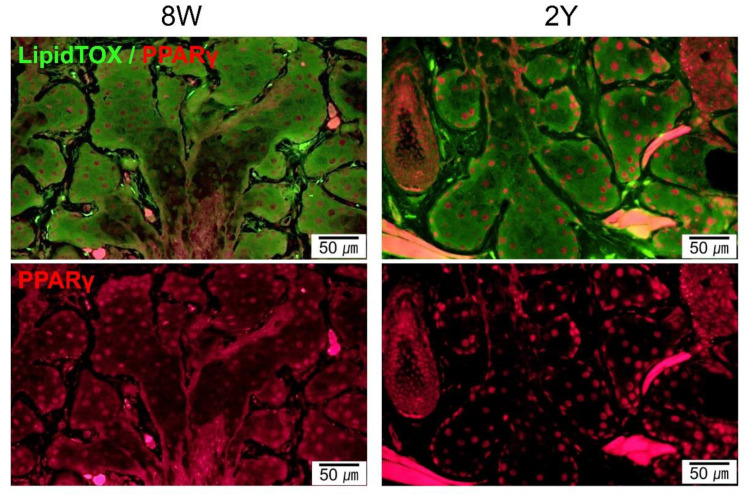
Representative images of coronal sections of the MGs stained with LipidTOX and PPARγ immunofluorescence (coronal section; 400×). Lipid droplets showed a similar distribution for 8W- and 2Y-old mice. PPARγ staining shows cytoplasmic and nuclear localization in 8W-old mice. In contrast, PPARγ staining shows predominant nuclear localization in 2Y-old mice.

MG dropout tended to increase with age (Figure 3A). The distribution of neutral lipids in the MGs did not seem to differ significantly with age (Figure 3B and Figure 4). It was found that PPARγ, which is known to regulate meibocyte differentiation and lipid synthesis, was distributed in both the cytoplasm and nucleus in 8W-old mice, but confined to the nucleus in 2Y-old mice (Figure 4). The number of Ki-67^+^ cells in the MGs as a marker for proliferating cells was significantly decreased in 1Y-old mice compared to 8W-old mice (Figure 3C; *p* = 0.036 for the upper eyelids by the Mann–Whitney test and 0.035 for the lower eyelids by the independent *t*-test). The number of CD45^+^ cells in the MGs as a marker for immune cells was not increased in 1Y-old mice relative to 8W-old mice (Figure 3D).

### 2.4. Aging-Dependent Inflammation Was Evident in the LGs

**Figure 5:**
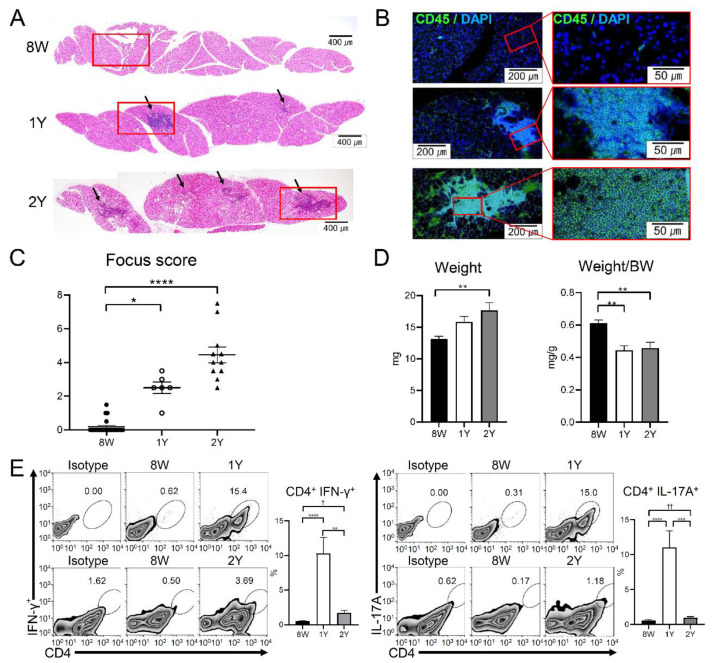
Representative images of the lacrimal gland (LG) sections acquired by (**A**) H&E staining (50×) and (**B**) CD45 immunofluorescence staining (100× and 400×). Arrows indicate lymphocytic infiltration foci. Red boxes of subfigure A are enlarged in subfigure B. (**C**) The inflammation focus score was higher in 1Y- and 2Y-old mice than in 8W-old mice (*p* = 0.010 and *p* < 0.0001, respectively; Kruskal–Wallis test followed by Dunn’s post hoc test; *n* = 22, 6, and 11 for 8W-, 1Y-, and 2Y-old mice, respectively). (**D**) LG weight was higher in 2Y-old mice than in 8W-old mice (*p* = 0.003; one-way ANOVA followed by Tukey’s post hoc test); however, the body weight (BW)-adjusted LG weight was lower in 2Y- and 1Y-old mice than in 8W-old mice (*p* = 0.002 and 0.001, respectively; one-way ANOVA followed by Tukey’s post hoc test; *n* = 8, 6, and 6 for 8W-, 1Y-, and 2Y-old mice, respectively). (**E**) After forward-scatter and side-scatter gating in the LGs, CD4^+^ IFNγ+ and CD4^+^ IL-17A^+^ cells were identified. The percentages of CD4^+^ IFNγ+ cells were higher in 1Y-old mice than in 8W- and 2Y-old mice (*p* < 0.0001 and *p* = 0.0015, respectively; one-way ANOVA followed by Tukey’s post hoc test). The percentages of CD4^+^ IL-17A^+^ cells were also higher in 1Y-old mice than in 8W- and 2Y-old mice (*p* < 0.0001 and *p* = 0.0004; one-way ANOVA followed by Tukey’s post hoc test). Although the percentages of CD4^+^ IFNγ^+^ cells and CD4^+^ IL-17A^+^ cells of 8W- and 2Y-old mice were not significantly different in a post hoc test (*p* = 0.785 and 0.975, respectively), there was a significant difference (*p* = 0.010 and *p* < 0.001, respectively) when an independent *t*-test was performed on only 8W- and 2Y-old mice. (*n* = 13, 6, and 3 for 8W-, 1Y-, and 2Y-old mice, respectively.) * *p* < 0.05, ** *p* < 0.01, and **** *p* < 0.0001 for the post hoc test, and ^†^ *p =* 0.010 and ^††^ *p <* 0.001 for the independent *t*-test. Data are presented as means ± standard error.

Cross-sections of the LGs showed ductal dilatation and periductal infiltration of inflammatory cells in 1Y- and 2Y-old mice (Figure 5A,B). The focus score of the LGs sequentially increased with aging and was significantly higher in 1Y- and 2Y-old mice than in 8W-old mice (Figure 5A–C; *p* = 0.010 and <0.0001, respectively; Kruskal–Wallis test followed by Dunn’s post hoc test). The weight of the LGs in 2Y-old mice was significantly higher than that of 8W-old mice (*p* = 0.003; one-way ANOVA followed by Tukey’s post hoc test). However, when the weight of the LGs was normalized by body weight, the LG density in aged mice (1Y- and 2Y-old) was significantly lower than that of 8W-old mice (Figure 5D; *p* = 0.002 and 0.001, respectively; one-way ANOVA followed by Tukey’s post hoc test). Flow cytometry analysis of the LGs revealed that the percentage of CD4^+^ interferon (IFN)-γ^+^ cells and CD4^+^ IL-17A^+^ cells increased in 1Y-old mice compared to 8W-mice (all *p* < 0.0001; one-way ANOVA followed by Tukey’s post hoc test), but decreased in 2Y-old mice (*p* = 0.0015 and 0.0004, respectively; one-way ANOVA followed by Tukey’s post hoc test) (Figure 5E). When an independent *t*-test was performed on only 8W- and 2Y-old mice, the percentages of CD4^+^ IFNγ^+^ cells and CD4^+^ IL-17A^+^ cells for 8W- and 2Y-old mice were significantly different (*p* = 0.010 and *p* < 0.001, respectively), although they were not significantly different in a post hoc test (*p* = 0.785 and 0.975, respectively).

### 2.5. Effector T Cells and Activated Antigen-Presenting Cells Were Increased in the Drainage Lymph Nodes of Aged Mice

**Figure 6:**
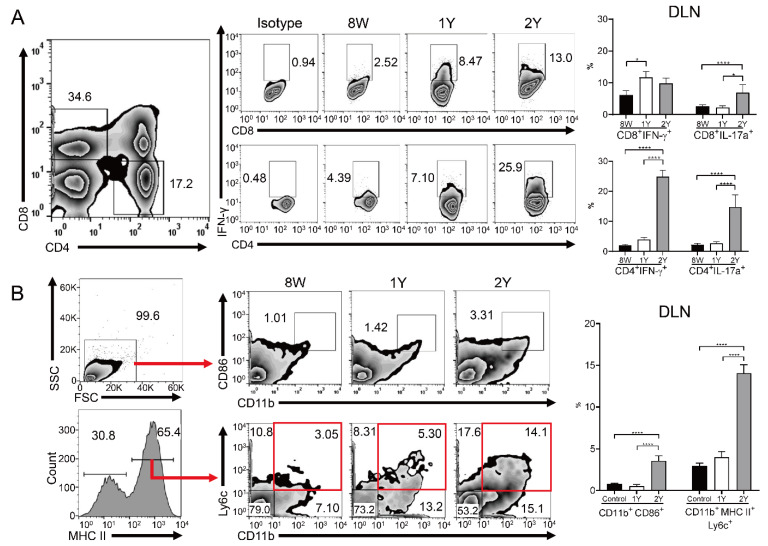
Flow cytometry for immune cells in the drainage lymph nodes (DLNs). (**A**) In DLNs, the percentage of CD8^+^ IFNγ^+^ T cells was higher in 1Y-old mice than in 8W-old mice (*p* = 0.032; Kruskal–Wallis test followed by Dunn’s post hoc test). The percentages of CD8^+^ IL-17A^+^, CD4^+^ IFN-γ^+^, and CD4^+^ IL-17A^+^ T cells in 2Y-old mice were significantly higher than in the other groups (one-way ANOVA). (**B**) The percentages of CD11b^+^ CD86^+^ and CD11b^+^ MHCII^+^ Ly6C^+^ cells were significantly higher in 2Y-old mice than in the other groups in the DLNs (one-way ANOVA). Red boxes indicate CD11b^+^ MHCII^+^ Ly6C^+^ cells. *n* = 15, 8, and 3 for 8W-, 1Y-, and 2Y-old mice, respectively. * *p* < 0.05 and **** *p* < 0.0001. Data are presented as means ± standard error.

Flow cytometry of drainage lymph nodes (DLNs) showed that the percentage of CD8^+^ IL-17A^+^, CD4^+^ IFN-γ^+^, and CD4^+^ IL-17A^+^ T cells was higher in 2Y-old mice than in 8W- and 1Y-old mice, with no significant differences between 8W- and 1Y-old mice (Figure 6A; *p* < 0.05; one-way ANOVA). The percentage of CD4^+^ and CD8^+^ IFNγ^+^ T cells was also increased in 1Y-old mice compared to 8W-old mice (Figure 6A; *p* < 0.05; one-way ANOVA). The percentages of the CD11b^+^ CD86^+^ and CD11b^+^ major histocompatibility complex (MHC)II^+^ Ly6C^+^ cells were significantly increased in 2Y-old mice compared to 8W- and 1Y-old mice (*p* < 0.05; one-way ANOVA), but equal for 8W- and 1Y-old mice (Figure 6B).

### 2.6. Oxidative Stress Increased in Both Glands of Aged Mice

**Figure 7:**
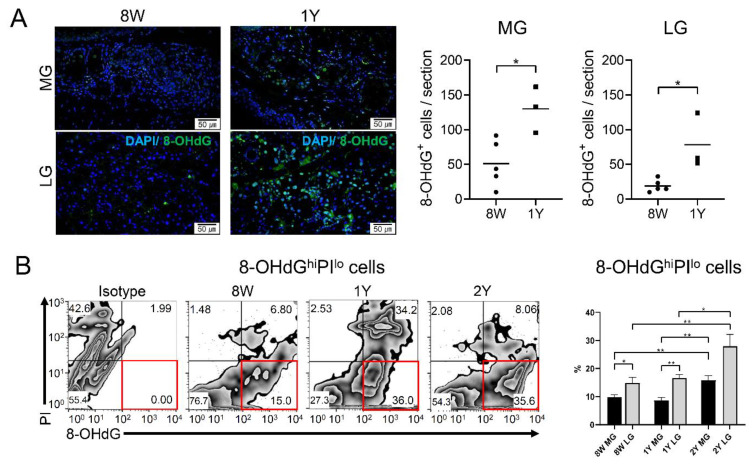
(**A**) Immunofluorescence staining of 8-OHdG in the MGs (sagittal section, upper panel) and LGs (lower panel; 400×). The number of 8-OHdG^+^ cells was significantly higher in 1Y-old mice (*n* = 5) than in 8W-old mice (*n* = 3) in both the MGs and LGs (*p* = 0.018 and 0.015, respectively; independent *t*-test). (**B**) The percentages of 8-OHdG^hi^ PI^lo^ cells were significantly higher in the LGs than in the MGs in 8W- and 1Y-old mice (*p* = 0.024 and 0.004, respectively; independent *t*-test). In both the MGs and LGs, 8-OHdG^hi^ PI^lo^ cells were significantly higher in 2Y-old mice than in 8W- and 1Y-old mice (one-way ANOVA; *n* = 9, 5, and 3 for 8W-, 1Y-, and 2Y-old mice, respectively). Red boxes indicate 8-OHdG^hi^ PI^lo^ cells. * *p* < 0.05 and ** *p* < 0.01. Data are presented as means ± standard error.

The number of 8-OHdG^+^ cells/section was higher in both glands of 1Y-old mice than in those of 8W-old mice (Figure 7A; *p* = 0.018 in the MGs and 0.015 in the LGs; independent *t*-test). In addition, the percentage of 8-OHdG^hi^ propidium iodide (PI)^lo^ cells was significantly higher in both glands of 2Y-old mice compared to those of 8W- and 1Y-old mice (Figure 7B; *p* = 0.009 and 0.007 in MGs, respectively, and *p* = 0.008 and 0.033 in LGs, respectively; one-way ANOVA followed by Tukey’s post hoc test). There were no differences between 8W- and 1Y-old mice in either gland. Surprisingly, when the oxidative stress of glands was compared at a given time point, the LGs always showed a higher percentage of 8-OHdG^hi^ PI^lo^ cells than the MGs, regardless of age (Figure 7B; *p* = 0.024 in 8W-old mice and *p* = 0.004 in 1Y-old mice; paired-*t* test)**.**

### 2.7. Senescence of Stem Cells Was Not Evident in Both Glands

**Figure 8:**
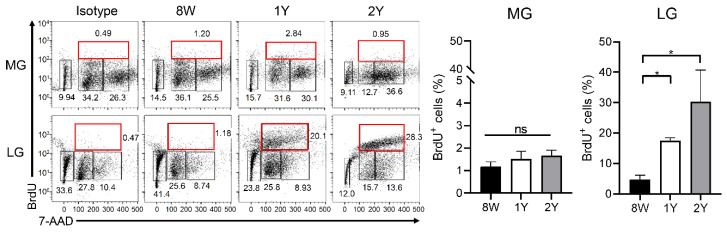
BrdU/7-AAD cell cycle analysis by flow cytometry. We excluded the apoptotic cells and gated 2n to 4n BrdU^+^ cells by 7-AAD gating. The percentage of BrdU^+^ (2n to 4n) cells in the MGs was not different among the groups (one-way ANOVA; *n* = 9, 5, and 3 for 8W-, 1Y-, and 2Y-old mice, respectively); however, the percentage of BrdU^+^ cells in the LGs was significantly higher in aged mice (1Y- and 2Y-old mice) than in 8W-old mice (*p* = 0.014 and 0.017, respectively; Kruskal–Wallis test followed by Dunn’s post hoc test; *n* = 9, 5, and 3 for 8W-, 1Y-, and 2Y-old mice, respectively). Red boxes indicate 2n to 4n BrdU^+^ cells. * *p* < 0.05. Data are presented as means ± standard error.

As label-retaining progenitors or S-phase cells, the percentage of BrdU^+^ cells during the cell cycle was not different in the MGs, depending on age. Interestingly, the percentage of BrdU^+^ cells in the LGs was significantly higher in aged mice (1Y- and 2Y-old mice) than in 8W-old mice (Figure 8; *p* = 0.014 and 0.017, respectively; Kruskal–Wallis test followed by Dunn’s post hoc test). When comparing the glands, the LGs tended to have a higher percentage of BrdU^+^ cells than the MGs, irrespective of age (*p* = 0.004 in 8W-old mice; Wilcoxon signed-rank test; *p* < 0.001 in 1Y-old mice; paired *t*-test).

## 3. Discussion

Our study showed that (1) dry eye and related ocular surface damage developed after one year of aging in a C57BL/6 male mouse model, (2) inflammation of the LGs was evident after one year of aging with a consistent increase of the focus score and CD4 ^+^ IFNγ^+^ cells, (3) atrophy of the MGs was evident after two years of aging, (4) oxidative stress was significantly increased in both glands after two years of aging, and (5) senescence of the stem cells was not evident in either gland. This suggests that aging-dependent dry eye may be related to the inflammation of LG, the development of atrophy of MG, and oxidative stress in both glands in this mouse model. The pathogenesis of age-related dry eye is a complex process. It is well-known that inflammation, senescence of the stem cells, atrophy of the functional cells, and oxidative stress in the LGs or MGs contribute to the pathogenesis of age-related DED [6]. However, it is not known how these factors affect age-related DED sequentially in the aging process. Therefore, our study is worthy of notice and may shed light on the pathogenesis of age-related dry eye by showing how the LGs and MGs deteriorate over time with regard to inflammation, stem cell properties, and oxidative stress.

**Figure A1:**
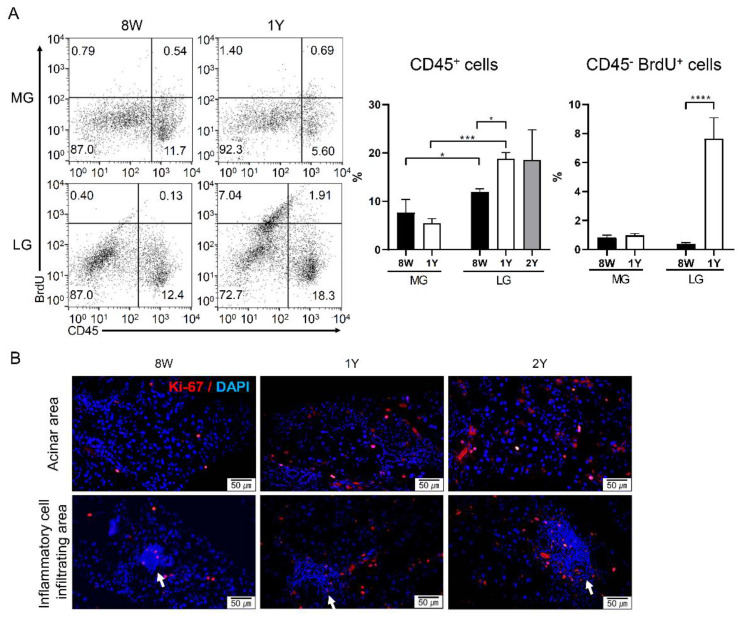
(**A**) CD45/BrdU gating by flow cytometry. The percentage of CD45^+^ cells in the LGs was significantly higher in 1Y-old mice than in 8W-old mice (*p* = 0.044; one-way ANOVA followed by Tukey’s post hoc test; *n* = 9, 5, and 3 for 8W-, 1Y-, and 2Y-old mice, respectively). The percentage of CD45^+^ cells in the LGs was also significantly higher than that in the MGs, irrespective of age (*p* = 0.019 in 8W-old mice and 0.0005 in 1Y-old mice, respectively; paired *t*-test; *n* = 5 per group). The percentage of CD45^−^ BrdU^+^ cells in the MGs was not different between 8W- and 1Y-old mice (*p* = 0.483); however, the percentage of CD45^−^ BrdU^+^ cells in the LGs was significantly higher in 1Y-old mice than in 8W-old mice (*p* < 0.0001; independent *t*-test; *n* = 5 per group). (**B**) Representative images of the LG sections by Ki-67 immunofluorescence staining (400×). Arrows indicate inflammatory foci. The expression of Ki67^+^ cells in both the acinar and inflammatory cell infiltrating areas tends to increase with aging. * *p* < 0.05, *** *p* < 0.001, and **** *p* < 0.0001. Data are presented as means ± standard error.

First, we evaluated age-related changes in the LGs. Given that tear and protein secretion are energy-requiring active procedures, the LGs appear to be highly susceptible to pathological aging. Emerging evidence suggests that the LGs undergo inflammation, atrophy, fibrosis, or ductal cell proliferation with aging in humans, as well as rodents [6,9,10,11]. Aging impairs the LGs by altering multiple mechanisms, such as neuroendocrine, immunological, apoptotic, protein secretion, and oxidative stress pathways [11]. Chronological changes in the LGs were reported in mice with mast cell infiltration, decreased peroxidase secretion, and lipofuscin accumulation at 8 to 12 months of age, followed by T and B lymphocytic infiltration, decreased innervation, decreased acetylcholine release, and structural changes up to 24 months of age [5,11,12]. Given the post-mortem changes in the LGs in humans, C57BL/6 mice can be used as a model to evaluate age-related DED, since C57BL/6 mice develop age-related dacryoadenitis, MG dysfunction, and corneal staining [5]. Interestingly, an aging-related predominance of IFN-γ-secreting CD4^+^IFNγ^+^ or CD4^+^IL17^+^ cells in the LGs may depend on the sex of the rodent [5,12]. This study corroborated the previous findings on age-related inflammatory changes in the LGs with a predominance of CD4^+^IFNγ^+^ cells in male mice. The focus score in the LGs continuously increased with time, and the level of CD4^+^IFNγ^+^ cells or CD4^+^IL17^+^ was higher in the LGs for up to 2 years compared to 8W-old mice, although the level of both cells decreased in 2Y-old mice when compared with 1Y-old mice. Meanwhile, the DLNs of 2Y-old mice showed an increase in CD4^+^IFNγ^+^ and CD4^+^IL17^+^ cell populations, along with an increased population of CD8^+^IL17^+^ cells, suggesting that both IFN-γ- and IL17-secreting cells are involved in age-related systemic autoimmune features, unlike the LGs. This finding may present different age-related changes of IL-17^+^ or IFNγ^+^ cells, depending on the location. A previous report also showed that the percentage of IFNγ^+^ cells increased across all tissues (conjunctiva, LG, and lymph nodes) at 24 months, while, on the contrary, the percentage of IL-17^+^ cells decreased in the LG and conjunctiva, unlike in the lymph nodes, at 24 months [4]. A healthy human study has demonstrated that age deeply impacts the contraction of CD4^+^IL-17^+^ cell populations in peripheral blood, while the population of CD4^+^IFNγ^+^ cells are relatively maintained, regardless of age [13]. In addition, the contraction rate differs depending on age, exhibiting a temporary increase of the percentage of CD4^+^IL-17^+^ cells in blood from those aged 35 to 49-years old, a decrease of the percentage of CD4^+^IL-17^+^ cells in the aged group (50 to 65-years old), and an increase of the percentage of CD4^+^IL-17^+^ cells again in the further-aged group (>65-years old) [14]. However, the secreting ability of IL-17 after in vitro stimulation with phytohemagglutinin continuously decreased, depending on age, owing to cellular senescence [14]. Given that the survival of immune cells may be affected differentially by the surrounding environment where the resident tissue cells provide survival factors, the shifting of IL-17^+^ cell or IFN γ^+^ cell changes from 1 year of age to 2 years of age is presumably affected by the (1) pathogenic increase of autoimmunity, (2) age-dependent cellular senescence, or (3) different survival rates based on where they reside. The changes in effector T cells were accompanied by increased populations of activated antigen-presenting cells in the DLNs, suggesting that an increased presence of the antigen is crucial to age-related dry eye, as previously reported [4]. High levels of IFN-γ in the conjunctiva are known to impact the loss of goblet cells [5,12]. Our study showed a decreased goblet cell density in 1Y-old mice, along with an increase in CD4^+^IFNγ^+^ cells in LG. However, there was no significant difference in the goblet cell density in 2Y-old mice compared to 8W-old mice, although the level of CD4^+^IFNγ^+^ cells in LG was still higher than that in young mice. We did not evaluate conjunctival Th1 cells directly, and this may have caused the difference between our results and those presented in a previous report [12]. Given that the relationship between goblet cell loss and aging remains controversial, depending on the detection method employed for goblet cells in humans [15,16], aging-related goblet cell changes should be further investigated. On the other hand, oxidative damage may be a key player in the pathogenesis of age-related and non-age-related dry eye [8,17,18], and our flow cytometric data were consistent with previous studies showing that 8-OHdG^+^ cells significantly increased in both the LGs and MGs of 2Y-old mice. This may support the study suggesting that calorie restriction can be a treatment option for age-related dry eye by reducing oxidative stress [19]. In 1Y-old mice, an inconsistency was observed between the histological findings and flow cytometric data. This occurrence might have been caused by an equivocal increase in 8-OHdG^+^ cells or by the semi-quantitative counting of histological sections with limited areas. Taken together, significant oxidative stress was evident in both the LGs and MGs after two years of age. Interestingly, the population of BrdU^+^ cells (label-retaining progenitor or S-phase cells) tended to increase over time in the LGs, while no significant changes in BrdU^+^ cells were observed over time in the MGs. When we gated out the CD45^+^ BrdU^+^ cells to remove a portion of the immune cells (Appendix A Figure A1A), the population of CD45^−^ BrdU^+^ cells still tended to increase. Given that BrdU^+^ label-retaining or S-phase cells can be observed in acini, duct, and myoepithelial cells, this change might have been caused by ductal or myoepithelial cell proliferation that occurs in aged mice or compensatory proliferative responses against inflammatory damaging signals in the LGs (Appendix A Figure A1B) [6,20]. One study reported that the BrdU^+^ label-retaining acinar cell population was 11.98 cells/mm^2^ in the LGs following a 2-week chase period after 7 days of consecutive BrdU injections [20]. We observed flow cytometric data. Therefore, a direct comparison with the histological data presented in the report mentioned above was not possible. The interpretation and data shape of the BrdU^+^ cells may differ, depending on the BrdU injection time. The longer the chase period after BrdU injection, the larger the population of slow-cycling progenitor cells that can be observed relative to the population of transient amplifying cells. In the <24 h interval between the observation and BrdU injection, the majority of BrdU^+^ cells indicated S-phase cells when gated together with 7-aminoactinomycin D (7-AAD). After two weeks of the chase period in our study, the BrdU^+^ cells included both progenitors and proliferating S-phase cells. Thus far, senescence of the progenitor cells was not evident in the LGs. However, the cells could not be further discriminated into acinar, ductal, or myoepithelial cells. A further investigation using acinar-specific or acinar-excluding markers is pending to confirm whether senescence of the acinar progenitor cells is significantly increased in aged LGs.

**Figure 9:**
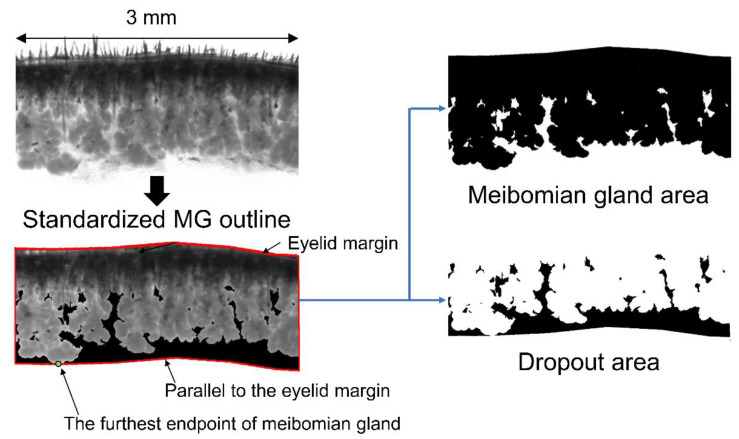
Ex vivo transillumination meibography images of the central 3-mm area of interest were analyzed. “The standardized MG outline” was defined with an imaginary line starting at the furthest endpoint of the MG and parallel to the eyelid margin. The area of the MG, including the eyelid margin, was defined as “the MG area”, and the area where the MG does not exist inside the standardized MG outline was defined as “the dropout area”.

Second, we assessed age-related morphological changes in the MGs. For highly qualified images of dropout, infrared transilluminated images of the MGs were taken after each eyelid was excised en bloc. A wide spectrum of white light was used as the light source, and an infrared transmitting filter was attached in front of the objective lens to obtain infrared images. As a result, compared to 8W-old mice, 1Y- and 2Y-old mice showed definite MG dropout as atrophy in the lower eyelid (Figure 2A). To quantify the degree of MG atrophy, the area of the MGs in the central 3 mm eyelid was measured (Figure 9). The increasing tendency of MG areas may be related to growth according to the aging process. Meanwhile, lower MG dropout areas increased sharply between 1 and 2 years (Figure 2B). This indicates that the lower MGs seem to be more affected by the aging process than the upper MGs. The result corroborates previous human studies suggesting increased dropout of the lower MGs relative to the upper MGs [21,22,23,24]. In addition, we found a negative correlation between the lower MG area and ocular surface staining, which is also a novel finding and supports clinically relevant dry eye changes with age. Further studies to confirm this outcome are in development.

To the best of our knowledge, this is the first study to quantify MG atrophy using mouse meibography. If we measure the MG area in a histological section of the eyelid, the MG area will vary, depending on the position of the section. Therefore, it seems best to measure the MG area with meibography, just like in humans. In human studies, the dropout area can be directly measured by meibography after eversion of the eyelid [25,26]. However, in this study, black pigmentation of the C57BL/6 mice still blocked the distal MG, even in the infrared image. Therefore, this ex vivo study requires an arbitrary reference line, such as the “standardized MG outline”. Although the quantification of dropout is quite a difficult process to achieve using mouse meibography, the dropout area could be measured inside the standardized MG outline. This study corroborated a human study by showing that atrophy increased with age by non-contact meibography [22,27]. It appears that atrophic changes affect the MGs slowly over time, and inflammatory changes affect the LGs at around one year.

Ki-67 is a nuclear antigen that is expressed during the cell cycle, present at stage G1, and used as a proliferation marker [28]. In this study, Ki-67 expression was lower in the acini of the MGs of 1Y-old mice compared to 8W-old mice (Figure 3C), which was consistent with a previous study showing that many Ki-67^+^ basal nuclei were present in the acini of MGs from young mice, but reduced in 1Y- to 2Y-old mice [28]. PPARγ is a member of the closely homologous genes ubiquitously expressed in various tissues and the major subtype expressed in sebocytes, where it regulates the expression of genes involved in lipogenesis [29,30]. In this study, the nuclear staining of PPARγ was similar in meibocytes between young and old mice, but the distribution of PPARγ staining in the cytoplasm was only noted in young mice (Figure 4) and correlated well with previous studies [28,31]. In summary, morphological atrophy increased over time with a decrease in the proliferation and differentiation of meibocytes. These changes were very similar in humans [7,31,32], which is a finding supported by this study.

In addition, we compared time-dependent inflammatory, oxidative, and proliferative capacity changes between the LGs and MGs. Regarding inflammation, the LGs presented more inflammation over time than the MGs based on the histological findings with CD45 staining (Figure 3 and Figure 5) or by comparative gating with CD45^+^ cells in flow cytometry (Appendix A Figure A1). Notably, the 8-OHdG^hi^ PI^lo^ cells in the LGs were significantly higher than in the MGs of both young mice and 1Y-old mice, suggesting that the LGs are susceptible to oxidative damage. This statistical difference between both glands disappeared after two years of age, at which point the population of 8-OHdG^+^ cells was equally high in both glands. The BrdU^+^ cell assay was used to detect label-retaining progenitors or S-phase cells. The senescence of these cells was not evident in both the LGs and MGs up to two years. There is an inconsistency between the histological counting with KI-67 staining and flow cytometric data with BrdU cells employed to evaluate the proliferative capacity in MGs. Although KI-67^+^ expression was decreased in histological sections at one year, the population of BrdU^+^ cells in flow cytometry was not different over the first two years. Different detection methods and the usage of different biomarkers may have caused the inconsistency. KI-67^+^ cells indicate proliferating cells, such as transient amplifying cells, while BrdU^+^ cells after the chase period indicate progenitors, as well as proliferating cells. Therefore, the proliferative capacity may be reduced, but the population of progenitors appears to be equal in the MGs. On the contrary, the LGs did not show any reduction in proliferative capacity, even with an exclusion of CD45^+^ BrdU^+^ cells (Figure 8 and Appendix A Figure A1). In summary, aging-related inflammation affects the LGs more than the MGs, and aging-related oxidative stress affects the LGs more than the MGs in the early period, but equally in later periods. Aging-related stem cell senescence was not evident in both the LGs and MGs.

Our study had several limitations. As small numbers of animals were used owing to the limited budget, certain analyses could not include all 8W-, 1Y-, and 2Y-old mice (Appendix A Table A1 and Table A2) and the analyses with obvious differences were not statistically significant (e.g., the percentage of BrdU^+^ cells in LG between 1-year-old and 2-year-old mice). In addition, it might be better to include data from 6-month-old mice. Given that structural changes or innervation changes of LG were not evident until 8 to 12-months old [11] and that the expression of either PPAR-γ or Ki67 of MG in 2-month-old mice was similar to that in 6-month-old mice [28], we only analyzed 8-week-old mice as representatives of young mice, and 1-year-old and 2-year-old mice. Second, although C57BL/6 mice are a valuable tool for assessing age-related dry eye, meibographic illustration in these mice is challenging due to dense pigmentation of the lid. Third, in our study, only male mice were used. Given that the female sex contributes differently to the phenotypes of age-related dry eye due to hormonal influences, our data on the MGs and LGs are different from those that would be obtained from female mice. It is known that goblet cell loss and corneal barrier disruption are less common in males than in females, and T cell infiltration in the LGs is greater in males than in females [12]. Inconsistencies in goblet cell data compared with previous studies may also be influenced by sex. Likewise, later atrophic changes in the MGs may be influenced by the male sex and hormone levels. Therefore, our data should be interpreted with a consideration of sex. In addition to CD4^+^ cells, CD20^+^ cells (B cells) play an important role in the pathogenesis of Sjögren’s syndrome [33]. In this study, we did not evaluate CD20^+^ cells. Further evaluation will be needed in future studies. Although PAS was only applied for goblet cell identification, based on the previous study’s protocol [12,34], additional nuclear counterstaining may be a better option for goblet cell identification. Finally, we used whole eyelid tissue for meibomian gland analysis with flow cytometry. We did our best to remove the skin and eyebrows, but the tissue may have still included skin epithelial cells or stromal fibroblasts because the specific markers for meibocytes are not yet known.

Nevertheless, to the best of our knowledge, this is the first report to (1) present comparative data on age-related changes in the LGs and MGs regarding dry eye and (2) quantify MG atrophy using mouse meibography. These findings may be helpful in understanding the pathophysiology of age-related dry eye.

## 4. Materials and Methods

The experimental protocol was approved on 29 May 2017 by the Institutional Animal Care and Use Committee of the Seoul National University Biomedical Research Institute (IACUC no. 17-0093-C1A1). Animal experiments were performed in accordance with the ARVO Statement for Use of Animals in Ophthalmic Vision and Research.

### 4.1. Animals

A total of 56 C57BL/6 male mice were used in this study. Twenty-nine 8W (young), sixteen 1Y (middle aged), and eleven 2Y (elderly) mice were included. The mice were bred in a specific pathogen-free facility at the Biomedical Research Institute of Seoul National University Hospital (Seoul, Korea), maintained at 22–24 °C with 55 ± 5% relative humidity, and given free access to food and water. The number of samples (eyes, LGs, and eyelids) used in each experiment and list of groups included in each experimental analysis are summarized in Appendix A Table A1 and Table A2, respectively.

### 4.2. Clinical Evaluation

The corneal staining score and tear secretion test were performed under anesthesia (using a mixture of zoletil and xylazine at a ratio of 1:3). The corneal staining scores were blindly assigned by a single experienced ophthalmologist (CH. Y.) according to the National Eye Institute (NEI) scoring scheme. After a drop of 0.25% fluorescein dye was applied to the conjunctival sac for 30 s, the ocular surface was gently washed with 1 mL of normal saline, and corneal staining was observed using a microscope (Olympus SZ61; Olympus Corporation, Tokyo, Japan) under cobalt blue illumination. For the tear secretion test, phenol red-impregnated cotton threads (FCI Ophthalmics, Pembroke, MA, USA) were inserted into the lateral canthus of mice for 60 s. The amount of secreted tears was determined by measuring the length of the wet thread in millimeters. The weights of LG were measured and divided by the body weight to adjust the aging-related confounding factor based on a previous study protocol [5,35,36,37], and tear secretion was also divided by body weight before comparison [5,38].

### 4.3. Histopathology

The whole eyeball and LGs were excised and fixed in formalin. The samples were sliced into 4 μm sections and subjected to hematoxylin-eosin (H&E), PAS, and immunohistochemical staining for CD45, Ki-67, LipidTOX, PPAR-γ, and 8-OHdG. The number of goblet cells was calculated as the sum of PAS-stained cells in the upper and lower eyelids under a microscope using a 200× objective (8W, *n* = 12; 1Y, *n* = 6; 2Y, *n* = 3). The inflammatory focus score in the LGs was measured by counting CD45^+^ inflammatory cells in a blinded manner (>50 inflammatory cells/focus = 1, 25–50 inflammatory cells/focus = 0.5) (8W, *n* = 22; 1Y, *n* = 6; 2Y, *n* = 11) [39]. The number of CD45^+^, Ki-67^+^, and 8-OHdG^+^ cells was counted in the medium-(200×; CD45^+^ and Ki-67^+^) or high-power fields (400×; 8-OHdG^+^) of two to three different sections from the same animal and averaged as cell counts/section. Each staining protocol was adapted from the method used in previous studies [32,40,41,42]. The following primary anti-mouse antibodies were used: CD45 (ab10558; Abcam, Cambridge, MA, USA), Ki-67 (Cat no. 14-5698-82; eBioscience, San Diego, CA, USA), LipidTOX (H34475; Invitrogen, Carlsbad, CA, USA), PPAR-γ (Ab59256; Abcam, Cambridge, MA, USA), and 8-OHdG (GTX41980; GeneTex, Irvine, TX, USA).

### 4.4. Transillumination Meibography of Mice

After euthanasia, both upper and lower eyelids were gently excised (8W, *n* = 27; 1Y, *n* = 6; 2Y, *n* = 16). The skin and muscles were removed from the excised eyelids as much as possible. The eyelids were carefully placed between two standard microscopic slides, and the slide was then placed over a wide spectrum white light source (LED plate), which was modified from the study of Reyes et al. [43]. The light passing through the eyelids was captured using a microscope (Olympus SZ61; Olympus Corporation) equipped with an infrared filter (Hoya R72; TOKINA Co. Ltd., Tokyo, Japan) and a Charge-Coupled Device (CCD) camera (acA1600-20 um; Basler Inc., Ahrensburg, Germany). A region of interest was identified in the central 3 mm area and analyzed using ImageJ software (Version 1.52a; NIH Image, Bethesda, MD, USA). The MG area, including the eyelid margin, was semi-automatically selected using the Wand tool (tolerance range of 5 to 30) of ImageJ (NIH Image). After defining the farthest point from the eyelid margin within the MG area, an imaginary line parallel to the eyelid margin was drawn at that point. As this ex vivo measurement method in mice is different from the in vivo method in humans, “the standardized MG outline” was defined with an imaginary line starting at the furthest endpoint of the MG and parallel to the eyelid margin. The area of the MG, including the eyelid margin, was defined as “the MG area” and the area where the MG does not exist inside the standardized MG outline was defined as “the dropout area” (Figure 9).

### 4.5. 5-Bromo-2′-Deoxyuridine (BrdU) Injection and Cell Cycle Analysis

For cell cycle analysis, we performed BrdU/7-AAD analysis with the FITC BrdU Flow Kit (Cat no. 559619; BD Pharmingen, San Diego, CA, USA). BrdU is a thymidine analogue, and 7-AAD binds to double stranded DNA by intercalating between base pairs in G-C-rich regions [44]. With this combination, flow cytometric analysis enables the enumeration and characterization of cells that are actively synthesizing DNA (BrdU incorporation) in terms of their cell cycle position (i.e., apoptotic, G0/1, S, or G2/M phase) [45,46].

To evaluate the proportion of progenitor cells in the MGs and LGs, mice (8W, *n* = 9; 1Y, *n* = 5; 2Y, *n* = 3) were intraperitoneally injected with 50 μg BrdU (51-2420KC; BD Pharmingen) per g body weight twice a day 14 and 13 days before sacrifice (four injections in total). After cell acquisition by forward and side scattering (FSC-A and SSC-A, respectively), we excluded the apoptotic cells by 7-AAD gating and gated progenitors (2n) or present proliferating cells synthesizing DNA in the S-phase of the cell cycle (2n to 4n BrdU^+^ cells) [47]. The cells were assayed using a FACSCanto flow cytometer (BD BioSciences, Mountain View, CA, USA). Data were analyzed using Flowjo software (version 10.6.1; Tree Star, Ashland, OR, USA).

### 4.6. Flow Cytometry for Immune Cells and Oxidatively Stressed Cells

The LGs (number of samples: 8W, *n* = 18; 1Y, *n* = 11; 2Y, *n* = 3), and cervical lymph nodes as DLN (number of mice: 8W, *n* = 15; 1Y, *n* = 8; 2Y, *n* = 3), were extracted and collected. Bilateral cervical lymph nodes were pooled for analysis. To obtain the MGs, the skin was peeled off and the eyebrows were removed as much as possible after excision of the upper and lower eyelids. For MG analysis, upper and lower samples of each eye were pooled (8W, *n* = 9; 1Y, *n* = 5; 2Y, *n* = 3). The proportion of IFN-γ and IL-17A in CD4^+^ and CD8^+^ T cells was assessed as a subset of effector T cells in the LGs and DLNs [48]. CD11b^hi^CD86^hi^ cells were gated as activated antigen-presenting cells, and MHCII ^hi^CD11b^hi^Ly6C^hi^ cells were gated as activated antigen-presenting monocytic cells in DLNs [39]. 8-OHdG^hi^/PI^lo^ cells were gated as oxidatively stressed cells in both MGs and LGs based on the methods described in previous studies [49].

To obtain cell suspensions, the collected LGs and DLNs and eyelids were minced between the frosted ends of two glass slides in media containing RPMI (WelGENE, Daegu, Korea), 10% fetal bovine serum, and 1% penicillin-streptomycin.

For intracellular IFN-γ and IL-17A staining, the cell suspensions were stimulated for 5 h with 50 ng/mL phorbol myristate acetate and 1 μg/mL ionomycin in the presence of GolgiPlug (BD Pharmingen). The cells were stained with the following antibodies: CD4-PE-Cy7 (Cat no. 25-0041; eBioscience), CD8-PerCP-Cy5.5 (Cat no. 45-0081; eBioscience), IFN-γ-FITC (Cat no. 11-7311; eBioscience), and IL17A-APC (Cat no. 17-7177; eBioscience).

For the antigen-presenting cells and oxidatively stressed cells, cell suspensions were collected and incubated for 30 min at 4 °C with fluorescein-conjugated anti-mouse antibodies as follows: CD11b-PE-Cy7 (Cat no. 25-0112; eBioscience), CD86-APC (Cat no.17-0862; eBioscience), MHC class II - FITC (Cat no. 11-5321; eBioscience), Ly6c–PerCp-Cy5.5 (Cat no. 45-5932, eBioscience), 8-OHdG (GTX41980; GeneTex), and PI (Cat no.51-66211E; BD Pharmingen).

The cells were assayed using a FACSCanto flow cytometer (BD BioSciences). Data were analyzed using Flowjo software (version 10.6.1; Tree Star).

### 4.7. Statistical Analysis

The Shapiro–Wilk test was used to test the normality of the data. Appropriate parametric (independent or paired *t*-test) or non-parametric (Mann–Whitney or Wilcoxon signed-rank test) statistical tests were used to compare the two groups. A one-way ANOVA followed by Tukey’s post hoc test or a Kruskal–Wallis test followed by Dunn’s post hoc test was used for a comparison of more than two groups. A partial correlation analysis was conducted for the MG parameters and corneal staining score after the removal of confounding age factors. Statistical analyses were performed using GraphPad Prism software (version 8.2.0; GraphPad Software, La Jolla, CA, USA). Differences were considered statistically significant at *p* < 0.05.

## 5. Conclusions

This study compared aging-dependent changes between the MGs and LGs in male C57BL/6 mice and it suggests that the inflammation of LG, the development of atrophy of MG, and oxidative stress in both glands may be involved in aging-dependent dry eye in this mouse model.

## Abbreviations

1Y	One-year-old
2Y	Two-year-old
7-AAD	7-aminoactinomycin D
8W	Eight-week-old
BrdU	Bromo-2′-deoxyuridine
BW	Body weight
CCD	Charge-Coupled Device
DED	Dry eye disease
DLN	Drainage lymph node
H&E	Hematoxylin-eosin
IFN	Interferon
LG	Lacrimal gland
MHC	Major histocompatibility complex
MG	Meibomian gland
NEI	National Eye Institute
PAS	Periodic acid-Schiff
PI	Propidium iodide

## Appendix A

**Table A1:** Number of samples in each experiment. The eyes, LGs, and eyelids for MGs that were used in each experiment were taken from 58 samples of twenty-nine 8-week-old mice, 32 samples from sixteen 1-year-old mice, and 22 samples from eleven 2-year-old mice.

	Experiment	Number of Samples		
		8W	1Y	2Y
Dry eye assessment	Corneal staining (eyes)	26	12	22
	Tear secretion (eyes)	36	22	22
	Conjunctival goblet cell count (eyes)	12	6	3
Gross examination	MG area analyzed by meibography	27	6	16
	LG weight	8	6	6
MG analysis	Histology			
	H&E staining (coronal section)	8	6	0
	H&E staining, and LipidTOX/PPARγ IHC (coronal section)	10	0	8
	Ki-67 and CD45 IHC (coronal section)	5	3	0
	8-OHdG IHC (sagittal section)	5	3	0
	Flow cytometry *			
	8-OHdG^hi^PI^lo^ and BrdU^+^	4	0	3
	8-OHdG^hi^PI^lo^, BrdU^+^, and CD45^+^	5	5	0
LG analysis	Histology			
	H&E staining and CD45 IHC	9	0	3
	H&E staining, CD45 IHC, and Ki-67 IHC	9	3	8
	H&E staining, CD45 IHC, and 8-OHdG IHC	4	3	0
	8-OHdG IHC	1	0	0
	Flow cytometry			
	CD4^+^ IFNγ^+^ and CD4^+^ IL-17A^+^	9	6	0
	8-OHdG^hi^PI^lo^, BrdU^+^, and CD45^+^	4	5	0
	CD4^+^ IFN-γ^+^, CD4^+^ IL-17A^+^, 8-OHdG^hi^PI^lo^, BrdU^+^, and CD45^+^	5	0	3
Secondary lymphoid organs	Flow cytometry of DLN ^†^ (mice)	15	8	3

* Upper and lower samples of each eye were pooled and analyzed. ^†^ Bilateral cervical lymph nodes were pooled for analysis. Abbreviations: W, week-old; Y, year-old; MG, meibomian gland; LG, lacrimal gland; H&E, hematoxylin-eosin; IHC, immunohistochemical staining; DLN, drainage lymph node.

**Table A2:** List of groups included in each experimental analysis.

	Experiment	Included Groups
Dry eye assessment	Corneal staining	8W, 1Y, and 2Y
	Tear secretion	8W, 1Y, and 2Y
	Conjunctival goblet cell count	8W, 1Y, and 2Y
Gross examination	MG area analyzed by meibography	8W, 1Y, and 2Y
	LG weight	8W, 1Y, and 2Y
MG analysis	Histology	
	H&E staining	8W, 1Y, and 2Y
	LipidTOX/PPARγ IHC (coronal section) *	8W and 2Y
	Ki-67 and CD45 IHC *	8W and 1Y
	8-OHdG IHC *	8W and 1Y
	Flow cytometry	
	8-OHdG^hi^PI^lo^	8W, 1Y, and 2Y
	BrdU^+^/7-AAD	8W, 1Y, and 2Y
LG analysis	Histology	
	H&E staining and CD45 IHC	8W, 1Y, and 2Y
	Ki-67 IHC	8W, 1Y, and 2Y
	8-OHdG IHC *	8W and 1Y
	Flow cytometry	
	CD4^+^ IFNγ^+^ and CD4^+^ IL-17A^+^	8W, 1Y, and 2Y
	8-OHdG^hi^PI^lo^	8W, 1Y, and 2Y
	BrdU^+^/7-AAD	8W, 1Y, and 2Y
Secondary lymphoid organs	Flow cytometry of DLN (mice)	8W, 1Y, and 2Y

* Experiments performed in only two groups. Abbreviations: W, week-old; Y, year-old; MG, meibomian gland; LG, lacrimal gland; H&E, hematoxylin-eosin; IHC, immunohistochemical staining; DLN, drainage lymph node.
